# Supplementary material for: A fault-intrusion-tolerant system and deadline-aware algorithm for scheduling scientific workflow in the cloud
Source: PeerJ Comput Sci. 2021 Nov 2;7:e747. doi: 10.7717/peerj-cs.747 (PMC8576554; doi:10.7717/peerj-cs.747)
Supplement: Supplemental Information 6 [file peerj-cs-07-747-s006.zip › Myproject1.1/docs/org/cloudbus/cloudsim/power/PowerDatacenterBroker.html]

PowerDatacenterBroker


JavaScript is disabled on your browser.


- Overview
- Package
- Class
- Use
- Tree
- Deprecated
- Index
- Help

- Prev Class
- Next Class

- Frames
- No Frames

- All Classes

- Summary:
- Nested |
- Field |
- Constr |
- Method

- Detail:
- Field |
- Constr |
- Method


org.cloudbus.cloudsim.power

## Class PowerDatacenterBroker

- java.lang.Object
- - org.cloudbus.cloudsim.core.SimEntity
  - - org.cloudbus.cloudsim.DatacenterBroker
    - - org.cloudbus.cloudsim.power.PowerDatacenterBroker

- All Implemented Interfaces:
  :   java.lang.Cloneable

  ---

    

  ```
  public class PowerDatacenterBroker
  extends DatacenterBroker
  ```

  A broker for the power package.
  If you are using any algorithms, policies or workload included in the power package please cite
  the following paper:
  Anton Beloglazov, and Rajkumar Buyya, "Optimal Online Deterministic Algorithms and Adaptive
  Heuristics for Energy and Performance Efficient Dynamic Consolidation of Virtual Machines in
  Cloud Data Centers", Concurrency and Computation: Practice and Experience (CCPE), Volume 24,
  Issue 13, Pages: 1397-1420, John Wiley & Sons, Ltd, New York, USA, 2012

  Since:
  :   CloudSim Toolkit 2.0

- - ### Field Summary

    - ### Fields inherited from class org.cloudbus.cloudsim.DatacenterBroker

      `cloudletList, cloudletReceivedList, cloudletsSubmitted, cloudletSubmittedList, datacenterCharacteristicsList, datacenterIdsList, datacenterRequestedIdsList, vmList, vmsAcks, vmsCreatedList, vmsDestroyed, vmsRequested, vmsToDatacentersMap`
    - ### Fields inherited from class org.cloudbus.cloudsim.core.SimEntity

      `FINISHED, HOLDING, RUNNABLE, WAITING`
  - ### Constructor Summary

    Constructors

    Constructor and Description

    `PowerDatacenterBroker(java.lang.String name)`

    Instantiates a new power datacenter broker.
  - ### Method Summary

    Methods

    | Modifier and Type | Method and Description |
    | --- | --- |
    | `protected void` | `processVmCreate(SimEvent ev)` Process the ack received due to a request for VM creation. |

    - ### Methods inherited from class org.cloudbus.cloudsim.DatacenterBroker

      `bindCloudletToVm, clearDatacenters, createVmsInDatacenter, finishExecution, getCloudletList, getCloudletReceivedList, getCloudletSubmittedList, getDatacenterCharacteristicsList, getDatacenterIdsList, getDatacenterRequestedIdsList, getVmList, getVmsAcks, getVmsCreatedList, getVmsDestroyed, getVmsRequested, getVmsToDatacentersMap, incrementVmsAcks, processCloudletReturn, processEvent, processOtherEvent, processResourceCharacteristics, processResourceCharacteristicsRequest, setCloudletList, setCloudletReceivedList, setCloudletSubmittedList, setDatacenterCharacteristicsList, setDatacenterIdsList, setDatacenterRequestedIdsList, setVmList, setVmsAcks, setVmsCreatedList, setVmsDestroyed, setVmsRequested, setVmsToDatacentersMap, shutdownEntity, startEntity, submitCloudletList, submitCloudlets, submitVmList`
    - ### Methods inherited from class org.cloudbus.cloudsim.core.SimEntity

      `cancelEvent, clone, getEventBuffer, getId, getName, getNextEvent, getNextEvent, getState, numEventsWaiting, numEventsWaiting, pause, run, schedule, schedule, schedule, schedule, scheduleFirst, scheduleFirst, scheduleFirst, scheduleFirst, scheduleFirstNow, scheduleFirstNow, scheduleFirstNow, scheduleFirstNow, scheduleNow, scheduleNow, scheduleNow, scheduleNow, selectEvent, send, send, send, send, sendNow, sendNow, sendNow, sendNow, setEventBuffer, setId, setState, waitForEvent`
    - ### Methods inherited from class java.lang.Object

      `equals, finalize, getClass, hashCode, notify, notifyAll, toString, wait, wait, wait`

- - ### Constructor Detail


    - #### PowerDatacenterBroker

      ```
      public PowerDatacenterBroker(java.lang.String name)
                            throws java.lang.Exception
      ```

      Instantiates a new power datacenter broker.

      Parameters:
      :   `name` - the name

      Throws:
      :   `java.lang.Exception` - the exception
  - ### Method Detail


    - #### processVmCreate

      ```
      protected void processVmCreate(SimEvent ev)
      ```

      **Description copied from class: `DatacenterBroker`**

      Process the ack received due to a request for VM creation.

      **Overrides:**
      :   `processVmCreate` in class `DatacenterBroker`

      Parameters:
      :   `ev` - a SimEvent object


- Overview
- Package
- Class
- Use
- Tree
- Deprecated
- Index
- Help

- Prev Class
- Next Class

- Frames
- No Frames

- All Classes

- Summary:
- Nested |
- Field |
- Constr |
- Method

- Detail:
- Field |
- Constr |
- Method
